# Characterization and Optimization of Polymer-Ceramic Pressure-Sensitive Paint by Controlling Polymer Content

**DOI:** 10.3390/s110706967

**Published:** 2011-07-04

**Authors:** Hirotaka Sakaue, Takuma Kakisako, Hitoshi Ishikawa

**Affiliations:** 1 Aerospace Research and Development Directorate, Japan Aerospace Exploration Agency, Chofu, Tokyo 182-8522, Japan; 2 Department of Mechanical Engineering, Tokyo University of Science, Chiyoda, Tokyo 102-0073, Japan; E-Mails: advmeas@chofu.jaxa.jp (T.K.); ishi@rs.kagu.tus.ac.jp (H.I.)

**Keywords:** pressure-sensitive paint, polymer ceramic, optimization

## Abstract

A pressure-sensitive paint (PSP) with fast response characteristics that can be sprayed on a test article is studied. This PSP consists of a polymer for spraying and a porous particle for providing the fast response. We controlled the polymer content (%) from 10 to 90% to study its effects on PSP characteristics: the signal level, pressure sensitivity, temperature dependency, and time response. The signal level and temperature dependency shows a peak in the polymer content around 50 to 70%. The pressure sensitivity was fairly constant in the range between 0.8 and 0.9 %/kPa. The time response is improved by lowering the polymer content. The variation of the time response is shown to be on the order of milliseconds to ten seconds. A weight coefficient is introduced to optimize the resultant PSPs. By setting the weight coefficient, we can optimize the PSP for sensing purposes.

## 1. Introduction

Pressure-sensitive paint (PSP) has been widely used in aerospace applications [1]. It uses a photophysical process of oxygen quenching to relate an oxygen pressure of a testing fluid to a luminescent signal. A PSP is composed of a luminophore and a supporting matrix. The former gives a luminescent signal and the latter holds the luminophore onto a testing article. A PSP can be categorized by the supporting matrix: polymer PSP and porous PSP. The supporting matrix greatly influences the response time of a PSP [2]. The former uses a polymer as a supporting matrix. Gaseous oxygen needs to permeate into this layer to cause the oxygen quenching. This limits the time response of this type of PSP on the order of seconds or sub-seconds. The latter uses a porous material as a supporting matrix. Gaseous oxygen can diffuse into a pore to cause oxygen quenching with a luminophore on the porous surface. The time response of this PSP is on the order of ten microseconds [2]. By using a porous PSP combined with a fast frame-rate camera, the PSP technique can be applied to global unsteady flow measurements [3]. However, the material of supporting matrix limits the application of a porous PSP. For example, anodized-aluminum pressure-sensitive paint (AA-PSP), which is the fastest PSP, is only applicable to aluminum [4]. There is a need to develop a fast PSP that can be sprayed onto any test article.

**Figure 1.**
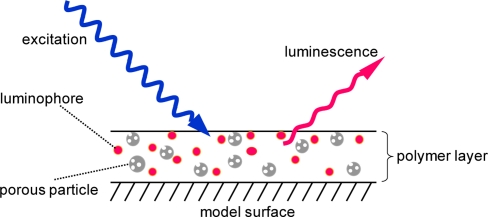
Schematic description of a polymer ceramic PSP (PC-PSP).

In this paper, a polymer ceramic PSP (PC-PSP), which provides a fast response characteristic and can be sprayed onto a testing article is studied [5]. The supporting matrix of this PSP is composed of a porous particle and a polymer (Figure 1). The former enhances the time response of the PSP, and the latter provides the spraying ability.

Some of the characterization results reported in the previous studies are summarized in Table 1. The pressure sensitivity, *σ*, was characterized from 0.21 to 0.95 %/kPa by changing the components of PC-PSP. The characterization of the temperature dependency, *δ*, was reported as −1.24 %/°C. However, neither the effects of the polymer to porous particle (polymer content) nor the PSP components were discussed. The response time, *τ*, ranged from 25 μs to 100 ms, and was related to the polymer content and the PSP thickness. Even though the signal level, *η*, is important for global pressure measurement, this feature was not reported. We controlled the ratio of the polymer content to relate this quantity to the PSP characterizations: the signal level, pressure sensitivity, temperature dependency, and response time. An optimization of these characteristics is also included in this paper.

## 2. Experiments and Methods

### 2.1. Materials

We chose a silica gel from Sigma-Aldrich as a porous particle. It has a mean particle size of 2 to 25 μm. We chose RTV from ShinEtsu Silicone as a polymer. To mix these components, we used dichloromethane as a solvent. The polymer-particle mixture was ultrasonicated for 20 min to reduce the aggregation of the particles, then it was spin-coated on a 10-mm square aluminum plate. We adjusted the thickness of the polymer ceramic coating as 10 ± 3 μm, which was measured by an eddy current apparatus. For each polymer content, three PSP samples were prepared to study the repeatability of the PSP preparation. We used bathophen ruthenium from GFS Chemicals as a luminophore. It was dissolved in dichloromethane to provide a 0.1 mM solution. A solution of 5 mL was spin-coated onto the polymer ceramic coatings. We varied the polymer content from 10 to 90% to study its effects on the PSP characteristics. A total of nine different polymer contents were prepared. We set the polymer content of 60% as a reference. The PC-PSP at this condition was denoted as PCPSP*.

### 2.2. Steady-State Characterization

**Figure 2.**
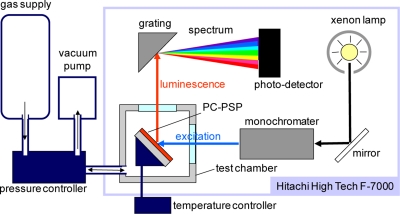
Schematic of the PC-PSP calibration setup.

Figure 2 schematically describes the steady-state characterization system, which consists of a spectrometer (Hitachi High Technologies, F-7000) and a pressure- and temperature-controlled chamber.

This system obtains the luminescent spectrum of a PC-PSP sample with varying pressures and temperatures. We characterized the signal level, pressure sensitivity, and temperature dependency from this system. The excitation wavelength was set at 460 nm. The luminescent intensity of a PC-PSP was determined by the integration of the spectrum within 620 ± 20 nm. The test gas was dry air. Throughout our characterizations, the reference conditions were 100 kPa and 25 °C. For detailed description of the system, refer Sakaue and Ishii [9].

For the signal level characterization, all the PC-PSP samples were measured with the same optical setup in the system but replacing samples in the chamber at the reference conditions. Based on Liu *et al*., the luminescent intensity, *I*, can be described by the gain of the photo-detector in a spectrometer, *G*, the emission from PC-PSP, *I_PCPSP_*, the excitation in the spectrometer, *I_ex_*, and the measurement setup component, *f_set_* [10]:

(1) $$I=G_{I_{\mathrm{PCPSP}}I_{\mathrm{ex}}}\;f_{\mathrm{set}}$$

In our setup, *G*, *I_ex_*, and *f_set_* were the same for all PC-PSP samples. We non-dimensionalized *I* by that of PCPSP*.We call this value as the signal level, *η*, shown in Equation (2):

(2) $$\eta =\frac{I}{I_{\mathrm{PCPSP}*}}(\%)$$

For the pressure calibration, the pressure, *P*, in the chamber was set from 5 to 120 kPa at a constant temperature at 25 °C. The luminescent intensity at the reference conditions, *I_ref_*, was used to derive *I_ref_/I*. This quantity can be related to pressures using the Stern-Volmer relationship [1]:

(3) $$\frac{I_{\mathrm{ref}}}{I}=A_{P}+B_{P}\cdot P$$

where *A_P_* and *B_P_* are calibration constants. A PSP with porous surface would show a non-linear relationship due to an oxygen adsorption on the porous surface [11]. Because a PC-PSP is a combination of a porous structure and a polymer, the same model describing a porous PSP would not be physically correct. As an alternative, we used the second-order polynomial to modify Equation (3):

(4) $$\frac{I_{\mathrm{ref}}}{I}=A_{P}+B_{P}\cdot P+C_{P}\cdot P^{2}$$

where *C_P_* is an additional calibration constant.

The pressure sensitivity, *σ*, describes the change in *I* over a given pressure change. This corresponds to a slope of Equation (4) at the reference conditions:

(5) $${\sigma =\frac{d(I_{\mathrm{ref}}/I)}{dp}|}_{p=p_{\mathrm{ref}}}=B_{P}+2C_{P}\cdot P_{\mathrm{ref}}\;(\%\text{kPa})$$

A PSP, in general, has a temperature dependency [1]. This influences *I*, which can be described as the second-order polynomial in Equation (6):

(6) $$\frac{I}{I_{\mathrm{ref}}}=A_{T}+B_{T}\cdot T+C_{T}\cdot T^{2}$$

where *A_T_*, *B_T_*, and *C_T_* are calibration constants. For the temperature calibration, the temperature, *T*, was set from 10 to 50 °C with a constant pressure at 100 kPa.

We defined the temperature dependency, *δ*, which is a slope of the temperature calibration at the reference conditions (Equation (7)). If the absolute value of *δ* is large, it tells us that the change in *I* over a given temperature change is also large. This is unfavorable condition as a pressure sensor. On the contrary, zero *δ* means that PC-PSP is temperature independent:

(7) $${\delta =\frac{d(I/I_{\mathrm{ref}})}{\mathrm{dT}}|}_{T=T_{\mathrm{ref}}}=B_{T}+2C_{T}\cdot T_{\mathrm{ref}}\;(\%/{}^{\circ}\text{C})$$

### 2.3. Unsteady-State Characterization

**Figure 3.**
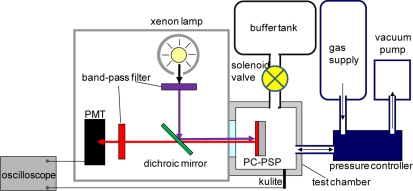
Schematic description of the unsteady-state calibration system.

The experimental setup of the unsteady-state characterization is shown in Figure 3. We used a step response apparatus consisting of a test chamber, buffer tank, and fast-acting solenoid valve. The valve connects the chamber and the buffer tank that creates a step change in pressure inside the chamber. A time delay from a step change of pressure was used to characterize the unsteady-state characterization as the time response [2]. We set the initial pressure of the chamber and the buffer tank at 100 and 1 kPa, respectively. The actual time to create a step pressure in the chamber was approximately 4 ms, which was measured by a high frequency response kulite sensor (XT-140-500). We used a continuous xenon lamp to excite a PC-PSP sample and a photo-multiplier tube (PMT) to collect *I* from the sample. The signals from the PMT and the kulite as a reference were sampled by a digital oscilloscope.

A step response of *I* was used to characterize the PC-PSP response time. It was calibrated to the pressure, *P*, by using the modified Stern-Volmer equation (Equation (4)), which is obtained from the steady-state characterization (Section 2.2). To cancel a variation by the calibration constants, normalized pressure, *P_norm_*, was derived:

(8) $$p_{\mathrm{norm}}=\frac{P-\text{min}\;P}{\text{max}\;P-\text{min}\;P}$$

where *min* and *max* denote the minimum and maximum values of a step change, respectively. We set the time the step pressure occurred as the initial time. Due to the limitations of the solenoid valve apparatus, the step change in *P* was at most 4 ms. We used the kulite sensor as a reference measurement to determine the response time. Assuming the kulite is fast enough to resolve the step change created by our setup, the response time, *τ*, was defined as the time difference between the kulite and the PC-PSP measurements. The time difference was determined at the time when *P_norm_* reaches from 100 to 10%:

(9) $$\tau =t_{\mathrm{PC}-\mathrm{PSP}}-t_{\mathrm{kulite}}$$

where *t_PC-PSP_* and *t_kulite_* denote the measurement times of PC-PCP and the kulite from the initial time to the time approaching at 10% of *P_norm_*.

## 3. Results and Discussion

### 3.1. PC-PSP Spectrum

**Figure 4.**
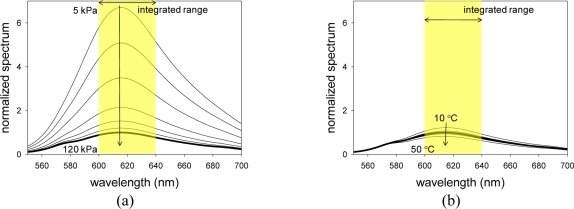
(**a**) Pressure spectra and (**b**) temperature spectra of PCPSP*. Thick line shows the spectrum at the reference conditions of 100 kPa and 25 °C.

Figure 4(a,b) show the luminescent spectra of PCPSP* with varying pressures and temperatures, respectively. Spectra were normalized by the luminescent peak under the reference conditions. We can see that with increase pressure, the luminescent spectrum decreased due to the oxygen quenching [1]. As the temperature increases, we can see the spectrum decrease due to the thermal quenching [1]. We can see that a luminescent peak exists around 620 nm. As described in Section 2.2, we integrated an obtained spectrum within 620 ± 20 nm to determine the luminescent intensity, *I*, for a given pressure and a temperature.

### 3.2. Signal Level

**Figure 5.**
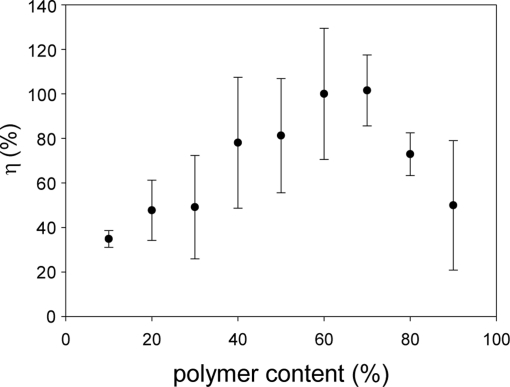
Relationship between the signal level, *η* (%), and the polymer content (%).

The signal level, *η*, was determined from Equation (2), which was shown in Figure 5.

The value of *η* was related to the polymer content. As a general trend, there was a peak that gave the maximum *η*. It would lie between 50 and 70% of the polymer content. Say that the minimum *η* was around 40%, more than a factor of two increase in *η* was obtained by controlling the polymer content. Due to a ±30% variation in the PC-PSP thickness that directly related to the surface area for applying the amount of luminophore, we saw relatively large error.

### 3.3. Pressure Sensitivity

**Figure 6.**
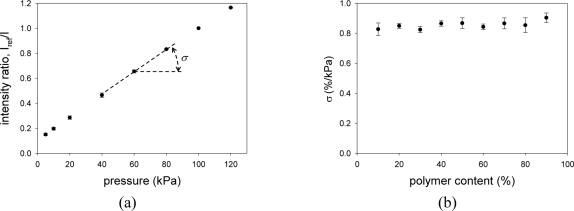
(**a**) The pressure calibration of PCPSP*. (**b**) Relationship between the pressure sensitivity, *σ* (%/kPa), and the polymer content (%).

Figure 6(a) shows the pressure calibration of PCPSP*. Calibration plots were fitted with Equation (4). A fairly linear relationship was seen. Even though a large error was seen in the signal level (Section 3.2), the ratio with the reference signal, *I_ref_/I*, greatly reduced the error. Figure 6(b) shows the pressure sensitivity, *σ*, related to the polymer content. It ranged from 0.8 to 0.9 %/kPa. Within the same luminophore used, our PC-PSP showed the highest *σ* (Table 1). It was higher than that of the fastest PSP (AA-PSP), which was 0.6 %/kPa [9]. The change in *σ* was smaller than that of *η* related to the polymer content.

### 3.4. Temperature Dependency

**Figure 7.**
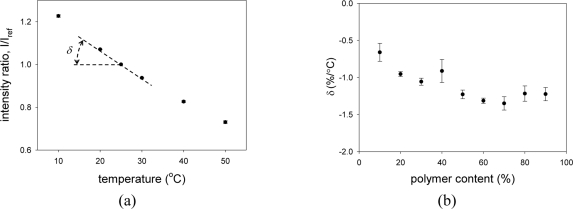
(**a**) The temperature calibration of PCPSP*. (**b**) Relationship between the temperature dependency, *δ* (%/°C), and the polymer content (%).

Figure 7(a) shows the temperature calibration of PCPSP*. Calibration plots were fitted with Equation (7). The calibrations showed a monotonic decrease in *I* with increase temperature. Similar to the pressure calibration, the ratio with the reference signal, *I/I_ref_*, greatly reduced the error. Figure 7(b) shows the temperature dependency, *δ*, related to the polymer content. As a general trend, it showed a peak at the polymer content between 50 and 70%. As a pressure sensor, *δ* is an undesirable quantity; zero *δ* is desirable. The results showed that the least temperature dependent PSP can be obtained by the polymer content of 10%. The reported *δ* of −1.24 %/°C was obtained at the polymer content of 3.5% (Table 1). If we extrapolate our results, our PC-PSP would show lower *δ* at this polymer content. Compared to *δ* of AA-PSP (−1.4 %/°C), the resultant PSP showed less temperature dependence [9].

### 3.5. Time Response

**Figure 8.**
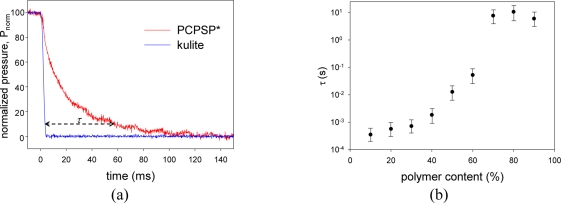
(**a**) Normalized pressure response of PCPSP*. (**b**) Relationship between the time response, *τ*(s), and the polymer content (%).

Figure 8(a) shows the step change results of PCPSP* and kulite. Figure 8(b) shows the time response of the resultant PSPs related to the polymer content. We can see that *τ* can be improved by lowering the polymer content. For a hundred milliseconds time scale, the step change in *P* of 4 ms is minimal. At the 10% of *P_norm_*, *τ* was determined. To consider that the step change of 4 ms is a minimal effect, *τ* over ten milliseconds would be valid. Even though a monotonic increase in *τ* was seen below ten milliseconds, we need to characterize *τ* by a faster step apparatus, such as a shock tube. The fastest PSP (AA-PSP) provides the response time on the order of ten microseconds [4]. Even if the result at 10% of the polymer content was valid, *τ* was still slower than that of AA-PSP. Based on the previous studies in Table 1, PC-PSP showed the response time of 25 μs at the PSP thickness of 5 μm. Our PC-PSP had the thickness of 10 ± 3 μm that would be one of the dominant factor to increase *τ* [2]. Based on the present results, a reduction of the polymer content up to 3.5% would be another important factor to improve *τ*. Referring to Kameda *et al*., we included the error bar as the square value of the thickness uncertainty: from 49% to 169% of determined *τ* [2]. We can see that a drastic change of *τ* was seen between 60 and 70% of the polymer content. Above 70% of the polymer content, *τ* was on the order of seconds to ten seconds.

### 3.6. Optimization of the Polymer Content

To discuss the effects on the polymer content, the characterization results were non-dimensionalized:

(10) $${\text{Signal level}:}^{\mathrm{norm}\;\eta =\frac{\eta -\eta_{\text{min}}}{\eta_{\text{max}}-\eta_{\text{min}}}}$$

(11) $${\text{Pressure sensitivity}:}^{\mathrm{norm}\;\sigma =\frac{\sigma -\sigma_{\text{min}}}{\sigma_{\text{max}}-\sigma_{\text{min}}}}$$

(12) $${\text{Temperature dependency}:}^{\mathrm{norm}\;\delta =\frac{\delta -\delta_{\text{min}}}{\delta_{\text{max}}-\delta_{\text{min}}}}$$

(13) $${\text{Response time}:}^{\mathrm{norm}\;\tau =\frac{\tau -\tau_{\text{max}}}{\tau_{\text{min}}-\tau_{\text{max}}}}$$

where the subscripts max and min denote the maximum and the minimum quantities, respectively. Here, the values of *δ* showed negative (Section 3.3). This means that *δ*_min_ is the most temperature dependent and *δ*_max_ the least temperature dependent. Therefore, higher the *normδ* gives less temperature dependent PC-PSP. On the other hand, *τ* is favorable if it has a smaller value. We non-dimensionalied *τ*, which was opposite to the other characterizations.

**Figure 9.**
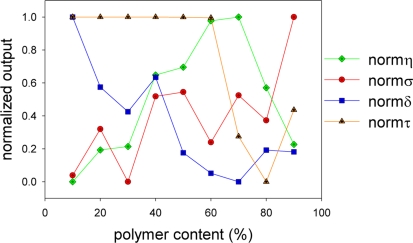
Normalized outputs of PC-PSPs related to the polymer content (%).

Figure 9 shows the non-dimensionalized characterizations of PC-PSPs. The maximum normalized outputs denote an optimum. Unfortunately, the same trend in all characterizations could not be seen. Thus, we could not determine the optimum from the normalized outputs. To determine an optimum of the polymer content, we introduce the weight coefficients, *α_η_*, *α_σ_*, *α_δ_*, and *α_τ_*. A sum of these coefficients is unity. We arbitrarily determine the importance of these coefficients depending on our sensing purposes. By using the weight coefficients, we determine an optimum value, *n_opt_*, as follows:

(14) $$n_{\mathrm{opt}}=\alpha_{\eta }\cdot \mathrm{norm}\eta +\alpha_{\sigma }\cdot \mathrm{norm}\sigma +\alpha_{\delta }\cdot \mathrm{norm}\delta +\alpha_{\tau }\cdot \mathrm{norm}\tau$$

Equation (14) tells us that the maximum *n_opt_* gives an optimum condition of the polymer content for given weight coefficients. If we design a PC-PSP such that all characterizations are equally important, we can set the weight coefficients as 1/4.

This condition is labeled as condition *1, and *n_opt_* are shown in Table 2. Under this weight condition, a PC-PSP with the polymer content of 40% gives an optimum. If we design a PC-PSP to optimize the response time and add an importance to the signal level, we can set *α_τ_* as 0.8, *α_η_* as 0.2, and others are 0. The value *n_opt_* was listed in Table 2 as condition *2. Under this condition, the polymer content of 60% gives an optimum. We included the condition *3 in Table 2, which designed the signal level, pressure sensitivity, and response time to be equally important. In this condition, *α_η_* = *α_σ_* =*α_τ_* = 0.3, and *α_σ_* = 0.1. An optimum can be given at the polymer content of 50%. This method does not provide the absolute optimum of the polymer content. However, it suggests us an optimum of the polymer content base on the sensing purposes.

## 5. Conclusions

We have introduced a pressure-sensitive paint (PSP) with fast response characteristics that can be sprayed. This PSP consists of a polymer for spraying and a porous particle for fast response. We varied the polymer content (%) to study its effects on PSP characteristics, such as the signal level, pressure sensitivity, temperature dependency, and time response. The signal level and temperature dependency showed a peak in the polymer content between 50 and 70%. By controlling the polymer content, the signal level was changed by more than a factor of two. The pressure sensitivity was fairly constant and ranged from 0.8 to 0.9 %/kPa. The time response was improved by lowering the polymer content. The variation of the time response on the order of milliseconds to ten seconds was seen. However, below the content of 40%, the time response approached the limitation of the apparatus. We used a weight coefficient to optimize the resultant PSPs. We could determine an optimum of the polymer content to provide an optimized PC-PSP for our sensing purposes.

## Figures and Tables

**Table 1.** Summary of PC-PSP characterizations in the previous studies.

**Luminophore/particle/polymer**	***σ***	***δ***	***τ***	**Comments**
RuDPP ^a^/TiO_2_^d^/B-1035	0.21 (%/kPa)	−1.24 (%/°C)	25 μs	polymer content: 3.5% [6]
				PSP thickness: 5 μm [7]
PtTFPP ^b^/TiO_2_^d^/B-1035	∼0.8 (%/kPa)	NA	61 μs to 100 ms	polymer content from 2.6 to 24% [8]
				PSP thickness not specified
RuDPP ^a^/alumina/B-1035	0.4 (%/kPa)	NA	NA	polymer content: 10% [8]
PtOEP ^c^/alumina/B-1035	0.95 (%/kPa)	NA	NA	polymer content: 10% [8]

a: bathophen ruthenium;

b: platinum (II) tetrakis(pentafluorophenyl)porphyrin;

c: platinum (II) octaethylporphine;

d: titanium dioxide.

**Table 2.** Optimum value, *n_opt_*, determined from weight coefficients, *α_η_*, *α_σ_*, *α_δ_*, and *α_τ_* for given polymer content (%). Condition *1: *α_η_* = *α_σ_* = *α_δ_* = *α_τ_* = 1/4. Condition *2: *α_τ_* = 0.8, *α_η_* = 0.2, and *α_σ_* = *α_τ_* = 0. Condition *3: *α_η_* = *α_σ_* = *α_τ_* = 0.3 and *α_δ_* = 0.1.

**Polymer content (%)**	*n_opt_* *1	*n_opt_* *2	*n_opt_* *3
10	0.51	0.79	0.60
20	0.52	0.80	0.64
30	0.41	0.77	0.60
40	0.70	0.84	0.94
50	0.60	0.86	0.95
60	0.57	0.91	0.88
70	0.44	0.67	0.61
80	0.28	0.49	0.34
90	0.46	0.54	0.66
